# Liver perfusion failure after pancreatoduodenectomy: clinical significance and perioperative risk factors

**DOI:** 10.1093/bjsopen/zrag056

**Published:** 2026-07-21

**Authors:** Mohammed Al-Saeedi, Julian M Deisenhofer, Hendrik B Sauer, Leonie Frank-Moldzio, Alina S Ritter, Tom Bruckner, Gabriel A Salg, Ali Ramouz, Philipp Mayer, Thomas Hank, Arianeb Mehrabi, Martin Loos, Thilo Hackert, Markus W Büchler, Oliver Strobel

**Affiliations:** Department of General, Visceral and Transplantation Surgery, Heidelberg University Hospital, Heidelberg, Germany; Department of General, Visceral and Transplantation Surgery, Heidelberg University Hospital, Heidelberg, Germany; Department of General, Visceral and Transplantation Surgery, Heidelberg University Hospital, Heidelberg, Germany; Department of General, Visceral and Transplantation Surgery, Heidelberg University Hospital, Heidelberg, Germany; Department of General, Visceral and Transplantation Surgery, Heidelberg University Hospital, Heidelberg, Germany; Institute of Medical Biometry and Informatics, Heidelberg University Hospital, Heidelberg, Germany; Department of General, Visceral and Transplantation Surgery, Heidelberg University Hospital, Heidelberg, Germany; Department of General, Visceral and Transplantation Surgery, Heidelberg University Hospital, Heidelberg, Germany; Department of Diagnostic and Interventional Radiology, Heidelberg University Hospital, Heidelberg, Germany; Department of General, Visceral and Transplantation Surgery, Heidelberg University Hospital, Heidelberg, Germany; Department of General, Visceral and Transplantation Surgery, Heidelberg University Hospital, Heidelberg, Germany; Department of General, Visceral and Transplantation Surgery, Heidelberg University Hospital, Heidelberg, Germany; Department of General, Visceral and Transplantation Surgery, Heidelberg University Hospital, Heidelberg, Germany; Department of General, Visceral and Transplantation Surgery, Heidelberg University Hospital, Heidelberg, Germany; Botton-Champalimaud Pancreatic Cancer Centre, Lisbon, Portugal; Department of General, Visceral and Transplantation Surgery, Heidelberg University Hospital, Heidelberg, Germany; Division of Visceral Surgery, Department of General Surgery, Medical University of Vienna, Vienna, Austria

**Keywords:** pancreatic surgery, hepatic perfusion, LPF, pancreatic cancer, coeliac axis stenosis, pancreatic fistula

## Abstract

**Background:**

The definition of liver perfusion failure (LPF) following pancreatic surgery and its perioperative risk factors remain unclear. This study aimed to define clinically significant LPF among patients undergoing partial pancreatoduodenectomy (PD) or total pancreatoduodenectomy (TP) and to identify perioperative risk factors associated with its incidence.

**Method:**

Patients undergoing partial PD/TP between 2014 and 2017 were identified from a prospectively maintained database. Various criteria for identifying LPF over time (that is, on postoperative days 1–4) were evaluated for clinical significance based on their association with liver-specific complications. Univariable and multivariable analyses were performed to determine the association of LPF with relevant outcome parameters and perioperative risk factors.

**Results:**

In the analysis of 815 patients, the optimal LPF model was identified as an increase in liver enzymes (alanine aminotransferase (ALT) and aspartate aminotransferase (AST)) ≥ 200 U/l for two consecutive days (LPF_2d). LPF severity was categorized as no LPF (AST/ALT < 200 U/l), mild (AST/ALT ≥ 200–< 500 U/l), moderate (AST/ALT ≥ 500–< 1000 U/l), and severe (AST/ALT ≥ 1000 U/l). In all, 81 patients were identified with LPF: 29 (3.6%), 29, and 23 with mild, moderate, and severe LPF, respectively. The occurrence of LPF was significantly associated with postoperative outcomes, including liver-specific complications, liver failure, need for radiological interventions, length of intensive care unit stay, and 90-day mortality. Multivariable analysis identified coeliac axis stenosis, arterial resection, and duration of the operation as independent risk factors for LPF.

**Conclusion:**

LPF is an underrated and serious postoperative complication in patients undergoing partial PD or TP. Based on the proposed definition (LPF_2d), LPF occurs in 9% of patients, is clinically relevant, and is associated with postoperative complications.

## Teaser

Liver perfusion failure (LPF) is a rare but serious complication following pancreatoduodenectomy. In this study, moderate/severe LPF was identified in 9.9% of 815 patients and was significantly associated with severe outcomes. Independent risk factors included coeliac axis stenosis, arterial resection, and a longer operative time. A prediction model was built to identify patients at high risk of LPF.

## Introduction

Due to significant advances in surgical techniques, preoperative assessment, and interdisciplinary patient care over past decades, there have been substantial improvements in outcomes for patients undergoing pancreatic surgery^1,2^. Overall morbidity in patients undergoing partial pancreatoduodenectomy (PD) and total pancreatoduodenectomy (TP) remains around 40%. With postoperative pancreatic fistula (POPF) accounting for 30% of the overall morbidity in patients undergoing partial PD^3,4^, increasing attention is being focused on other complications not predominantly related to the pancreas^5–8^.

Liver perfusion failure (LPF), characterized by elevated levels of alanine aminotransferase (ALT) and aspartate aminotransferase (AST), is a known complication in liver and transplantation surgery^9–11^. Recent studies have shown that LPF also occurs in patients undergoing partial PD or TP^5,12–14^. LPF results in ischaemic and hypoxic damage to the hepatocytes, leading to impaired liver function and an increased risk of liver failure after pancreatic resection^15–17^. Ischaemic liver complications may progress to necrotic liver tissue with abscess formation and the risk of superinfection. In the case of severe complications, these conditions trigger systemic infections, potentially ending in sepsis and multiorgan failure^16,17^. Despite extensive studies in liver surgery, the understanding of LPF in pancreatic resections remains unclear^5,18^.

The aim of this study was to establish a comprehensive definition of clinically relevant LPF following partial PD or TP, based on its association with liver-specific complications. The study sought to assess the association of LPF with other postoperative outcomes and to identify perioperative risk factors for LPF.

## Methods

### Study design and patient cohort

This study is based on an analysis of prospectively collected and retrospectively evaluated clinical information of an institutional database of the Heidelberg University Hospital containing information from consecutive pancreatic operations. The study was approved by the institutional ethics committee of the Heidelberg University Hospital (S-011/2015) and follows the STROBE guidelines for observational studies^19^.

All consecutive patients undergoing partial PD or TP between January 2014 and December 2017 at the Department of General, Visceral and Transplantation Surgery, Heidelberg University Hospital, were screened for inclusion in the study. Patients without preoperative contrast-enhanced computed tomography (CT) and patients with missing laboratory data were excluded from analysis. Patients with known advanced chronic liver disease or liver cirrhosis were not considered candidates for partial PD or TP and were therefore excluded from surgery. During the study period, neoadjuvant therapy was not routinely administered to patients with borderline-resectable disease and upfront resection was therefore the standard approach. The study cohort was not restricted to pancreatic ductal adenocarcinoma and included other tumour entities.

### Data collection

Clinical parameters extracted from the prospectively maintained database included age, sex, body mass index, American Society of Anesthesiologists grade, alcohol consumption, steatohepatitis, hepatitis, and smoking. Alcohol abuse was defined as clinically significant distress or functional impairment occur within a 12-month period. To assess the influence of previous upper abdominal surgery on the occurrence of LPF, data on previous procedures on the pancreas, liver, and stomach were collected. Data on preoperative biliary drainage (including endoscopic retrograde cholangiopancreatography, bile duct stenting, percutaneous transhepatic biliary drainage and T-tube drainage) and neoadjuvant chemotherapy or radiotherapy were extracted from the database.

Surgery-related parameters were also analysed, including the indication for the surgery performed, the type of pancreatic resection, extended and vascular resections^20^, blood loss, and the duration of the operation. To ensure a comprehensive approach to liver perfusion, variants of the hepatic blood supply and the coeliac trunk in terms of aberrant and accessory hepatic vessels^21^ were analysed. The impact of pre-existing compression of the coeliac trunk was reviewed by scanning the available CT data for coeliac axis stenosis (CAS) following a modified classification of Sugae *et al*.^22^. The presence of CAS was evaluated on CT scans during the arterial phase. The degree of stenosis was primarily assessed using sagittal reconstructions. Postoperative complications were categorized according to the Clavien-Dindo claasification^23^. Other postoperative complications, including POPF^3^, postpancreatectomy haemorrhage (PPH)^24^, and postoperative bile leakage^25^, were classified according to the definitions of the International Study Group of Pancreatic Surgery. All further postoperative radiological, endoscopic, and surgical interventions were recorded. In addition, the length of hospital stay, the length of intermediate care unit and intensive care unit stay, and the 90-day mortality rate were recorded.

Liver-specific complications were defined as liver failure and parenchymal liver damage or necrosis. Liver failure was classified according to the criteria of the International Study Group of Liver Surgery, which includes elevated serum bilirubin and/or international normalized ratio of prothrombin time levels in the postoperative period^15^. Parenchymal damage was recorded only in patients with radiologically confirmed ischaemic changes on CT scans, such as hypoenhanced or hypodense areas consistent with hepatic injury. Perihepatic collections were not categorized as liver-specific complications unless clearly associated with biliary leakage or intrahepatic necrosis.

### Identification of LPF and assessment of risk factors

To identify patients with LPF, the AST and ALT levels during the first 10 days after surgery were analysed. The previous definition of LPF, namely elevated AST/ALT levels on 1 day (LPF_1d)^5^, was tested, alongside three new models. LPF_1d was defined as peak serum ALT and AST levels on any of postoperative day (POD) 1–3. In this model, mild, moderate, and severe increases in peak serum ALT and AST concentrations on POD1–3 were defined as levels of < 500–250, 500–1000, and > 1000 units/l, respectively. Because previous studies indicated that short-term changes in liver enzymes do not necessarily correlate with postoperative complications^26–28^ , additional models with prolonged durations of elevations in liver enzymes over several consecutive days were tested, as follows:

- LPF_2d, in which LPF was defined as an ALT/AST elevation > 200 U/l for two consecutive postoperative days
- LPF_3d, in which LPF was defined as an ALT/AST elevation > 200 U/l for three consecutive postoperative days, with severity of perfusion classified as for LPF_2
- LPF_4d, in which LPF was defined as an ALT/AST elevation > 200 U/l for four consecutive postoperative days, with severity of perfusion classified as for LPF_2.

In all cases, the severity of LPF was graded according to the highest AST or ALT serum concentration within the observation period (2, 3, or 4 days) as no LPF (AST/ALT < 200 U/l), mild LPF (AST/ALT 200–499 U/l), moderate LPF (AST/ALT 500–999 U/l), or severe LPF (AST/ALT ≥ 1000 U/l).

To identify perioperative risk factors for postoperative LPF, the definitions of LPF were first tested for their clinical relevance by correlating them to postoperative outcome parameters, as well as liver-specific complications. Preoperative ALT levels were analysed in the entire cohort as a baseline variable. Subsequently, a sensitivity analysis was conducted in which all patients with preoperative ALT ≥ 500 U/l were excluded to ensure that these patients would not bias the tested definitions of LPF.

### Statistical analysis

IBM SPSS Statistics version 25.0 (IBM, Armonk, NY, USA) and GraphPad PRISM version 8 (GraphPad Software, San Diego, CA, USA) were used for statistical analyses. Two-tailed *P* < 0.05 was considered significant. Continuous data are presented as the mean with standard deviation or median and interquartile range and categorical data are presented as frequencies with percentages. Univariable analyses were performed using the Kruskal–Wallis test for continuous data and the χ^2^ test or Fisher's exact test for categorical data.

To identify independent predictors of LPF, variables with *P* < 0.1 in the initial univariable analysis were included in a multivariable binary logistic regression model. To avoid overfitting and address potential collinearity, variable selection was based on clinical relevance. Patients with missing values were excluded from the multivariable analyses. Results are reported as odds ratios (OR) with the corresponding 95% confidence interval (c.i.).

## Results

### Demographic and preoperative data

In all, 1429 patients underwent partial PD or TP between 2014 and 2017 at the Department of Surgery at Heidelberg University Hospital (*Fig. S1*). Of these patients, 511 (35.8%) met the exclusion criteria and were removed from the study. The mean age of the remaining 918 patients available for analysis was 64 years, and 515 (56.1%) were male (*Table 1*). Most patients (818, 89.4%) underwent upfront surgery, whereas 100 (10.9%) received neoadjuvant (radio-) chemotherapy. Preoperative jaundice was observed in 286 patients (31.2%). Among these patients, 280 (30.5%) underwent endoscopic retrograde cholangiopancreatography with bile duct stenting in 219 (23.9%); 21 patients (2.3%) received percutaneous transhepatic cholangiodrainage; and 12 patients (1.3%) underwent surgical bile duct drainage. No significant correlation was observed between preoperative biliary drainage and LPF, except for a significantly (*P* = 0.026) higher rate of T-tube drainage insertion in patients with mild and moderate LPF. Of the 918 patients, 687 (74.8%) underwent partial PD and 231 (25%) underwent TP. Additional organ resection was performed in 174 patients (19.0%), and 284 patients (30.9%) underwent vascular resections. Radiological screening of the available CT data revealed the presence of CAS in 256 patients (27.9%).

**Table 1:** Demographic and perioperative data of the entire cohort and according to the presence and degree of LPF_2d

	Total (*n* = 918)	LPF_2d				P*
		None (*n* = 808)	Mild (*n* = 48)	Moderate (*n* = 35)	Severe (*n* = 27)	
Age (years), mean(s.d.)	64(12)	64(12)	64(13)	64(12)	66(11)	0.706†
BMI (kg/m^2^), mean(s.d.)	25.4(4.4)	25.4(4.4)	25.7(3.8)	24.7(5.4)	27.8(6.3)	0.281†
**Sex**						
Female	403 (43.9%)	351 (43.4%)	25 (52.1%)	16 (45.7%)	11 (40.7%)	0.447‡
Male	515 (56.1%)	457 (56.6%)	23 (47.9%)	19 (54.3%)	16 (59.3%)	
Smoking	247 (26.9%)	226 (28.2%)	8 (16.7%)	7 (20.0%)	6 (23.1%)	0.050‡
Alcohol abuse	150 (16.5%)	136 (17.0%)	3 (6.3%)	2 (5.7%)	9 (34.6%)	0.273‡
**ASA grade**						
I	20 (2.3%)	16 (2.1%)	2 (4.4%)	2 (6.1%)	0 (0.0%)	0.088‡
II	491 (56.2%)	435 (56.4%)	26 (57.8%)	18 (54.5%)	12 (48.0%)	
III	355 (40.6%)	315 (40.9%)	17 (37.8%)	12 (36.4%)	11 (44.0%)	
IV	8 (0.9%)	5 (0.6%)	0 (0.0%)	1 (3.0%)	2 (8.0%)	
**Hepatitis**						
Hepatitis A	1 (0.1%)	1 (0.1%)	0 (0.0%)	0 (0.0%)	0 (0.0%)	0.987
Hepatitis B	3 (0.3%)	3 (0.4%)	0 (0.0%)	0 (0.0%)	0 (0.0%)	0.938
Hepatitis C	5 (0.5%)	5 (0.6%)	0 (0.0%)	0 (0.0%)	0 (0.0%)	0.877
Other	5 (0.5%)	3 (0.4%)	1 (2.1%)	0 (0.0%)	1 (3.7%)	0.052
Steatohepatitis	36 (3.9%)	34 (4.2%)	1 (2.1%)	0 (0.0%)	1 (3.7%)	0.564
Liver tumour/metastases	38 (4.1%)	36 (4.5%)	1 (2.1%)	0 (0.0%)	1 (3.7%)	0.524
Liver infiltration	5 (0.5%)	5 (0.6%)	0 (0.0%)	0 (0.0%)	0 (0.0%)	0.877
Other liver diseases	31 (3.4%)	25 (3.1%)	1 (2.1%)	3 (8.6%)	2 (7.4%)	0.197
Preoperative icterus	286 (31.2%)	241 (29.8%)	22 (45.8%)	13 (40.0%)	9 (33.3%)	0.078
Cholestasis	206 (22.4%)	179 (22.2%)	12 (25.0%)	8 (22.9%)	7 (25.9%)	0.938
**Preoperative biliary drainage**						
ERCP	280 (30.5%)	250 (30.9%)	18 (37.5%)	5 (14.3%)	7 (25.9%)	0.122
Bile duct stent	219 (23.9%)	201 (24.9%)	8 (16.7%)	4 (11.4%)	6 (22.2%)	0.180
PTBD	21 (2.3%)	19 (2.4%)	0 (0.0%)	1 (2.9%)	1 (3.7%)	0.698
T-tube drainage	12 (1.3%)	8 (1.0%)	2 (4.2%)	2 (5.7%)	0 (0.0%)	0.026
**Neoadjuvant therapy**						
None	818 (89.1%)	721 (89.3%)	42 (91.3%)	32 (91.4%)	23 (85.2%)	0.858
Radiotherapy	14 (1.5%)	11 (1.4%)	1 (2.1%)	2 (5.7%)	0 (0.0%)	0.191
**Chemotherapy**	121 (13.2%)	108 (13.4%)	5 (10.5%)	4 (11.5%)	4 (14.8%)	0.919
FOLFIRINOX	74 (8.1%)	66 (8.2%)	3 (6.3%)	1 (2.9%)	4 (14.8%)	0.367
Gemcitabine and paclitaxel	14 (1.5%)	14 (1.7%)	0 (0.0%)	0 (0.0%)	0 (0.0%)	0.586
Gemcitabine	10 (1.1%)	8 (1.0%)	0 (0.0%)	2 (5.7%)	0 (0.0%)	0.049
Other	23 (2.5%)	20 (2.5%)	2 (4.2%)	1 (2.9%)	0 (0.0%)	0.739
Chemoradiotherapy	12 (1.3%)	9 (1.1%)	1 (2.1%)	2 (5.7%)	0 (0.0%)	0.162‡
**Type of resection**						
Partial PD	687 (74.8%)	625 (77.4%)	29 (60.4%)	18 (51.4%)	15 (55.6%)	< 0.001‡
TP	231 (25.2%)	183 (22.6%)	19 (39.6%)	17 (48.6%)	12 (44.4%)	< 0.001‡
Vascular resection (total)	284 (30.9%)	219 (27.1%)	25 (52.1%)	25 (71.4%)	15 (55.6%)	< 0.001‡
**Arterial resection**	32 (3.5%)	16 (2.0%)	3 (6.3%)	5 (14.7%)	8 (29.6%)	< 0.001‡
Hepatic artery	18 (2.0%)	8 (1.0%)	1 (2.1%)	4 (11.4%)	5 (18.5%)	< 0.001
Right hepatic artery	4 (0.4%)	3 (0.4%)	0 (0.0%)	1 (2.9%)	0 (0.0%)	0.162
Left gastric artery	2 (0.2%)	1 (0.1%)	0 (0.0%)	1 (2.9%)	0 (0.0%)	0.008
Superior mesenteric artery	10 (1.1%)	10 (1.2%)	0 (0.0%)	0 (0.0%)	0 (0.0%)	0.711
Truncus coeliac	16 (1.7%)	6 (0.7%)	2 (4.2%)	2 (5.7%)	6 (22.2%)	< 0.001
**Venous resection**	252 (27.5%)	203 (25.2%)	22 (45.8%)	20 (57.1%)	7 (25.9%)	< 0.001‡
Portal vein	208 (22.7%)	165 (20.4%)	20 (41.7%)	17 (48.6%)	6 (22.2%)	< 0.001
Superior mesenteric vein	73 (8.0%)	60 (7.4%)	4 (8.3%)	7 (20.0%)	2 (7.4%)	0.064
Splenic vein	6 (0.7%)	5 (0.6%)	1 (2.1%)	0 (0.0%)	0 (0.0%)	0.586
Portal confluence	14 (1.5%)	10 (1.2%)	2 (4.2%)	2 (5.7%)	0 (0.0%)	0.066
Inferior vena cava	11 (1.2%)	9 (1.1%)	1 (2.1%)	0 (0.0%)	1 (3.7%)	0.528
Operation time (min), mean(s.d.)	338(86)	331(82)	374(94)	367(75)	418(123)	< 0.001
Blood loss (ml), mean(s.d.)	1269(1071)	1222(1042)	1400(1116)	1727(1043)	1870(1559)	< 0.001

Values are *n* (%) unless otherwise stated. *P* values tested the rate of different grades of LPF_2d for each parameter. LPF_2d, liver perfusion failure (alanine aminotransferase/aspartate aminotransferase elevation > 200 U/l) on two consecutive days; s.d., standard deviation; BMI, body mass index; ASA, American Society of Anaesthesiologists; ERCP, endoscopic retrograde cholangiopancreatography; PTBD, percutaneous transhepatic biliary drainage; FOLFIRINOX, folinic acid, fluorouracil, irinotecan, oxaliplatin; PD, pancreatoduodenectomy; TP, total pancreatoduodenectomy; min, minutes. *P values calculated using Chi-square test, except †Students *t* test and ‡χ^2^ test.

### Establishment of a LPF definition

To establish a comprehensive and clinically relevant definition of LPF, four models were tested in the cohort for their association with liver-specific complications. The overall LPF rates based on LPF_1d, LPF_2d, LPF_3 and LPF_4 were 19.2, 12, 7.3 and 4.8%, respectively (*Table S1*). Extending the AST/ALT observation period from POD1 up to POD4 resulted in decreased detection of mild (from 6.5 to 0.5%) and moderate (from 10.4 to 2.2%) LPF. Severe forms of LPF were consistently detected with all proposed models, with the highest percentage detected with the LPF_2d model (2.9%).

**Fig. 1:**
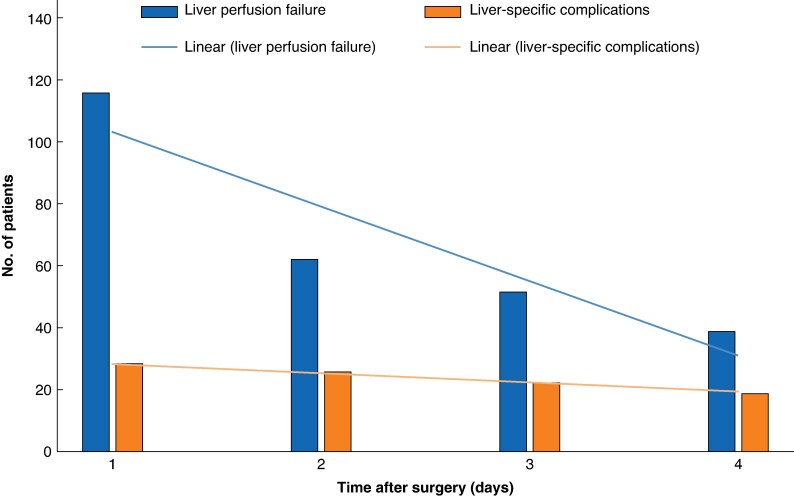
Incidence of liver perfusion failure and liver-specific complications over time Bar graphs show the number of patients experiencing moderate and severe liver perfusion failure and liver-specific complications on postoperative days 1, 2, 3, and 4.

To identify the best definition based on the clinical significance of LPF, the association of liver-specific complications with LPF and its severity levels within the different LPF models were assessed (*Table S2*). In this analysis, the associations between liver-specific postoperative complications and moderate and severe changes in AST/ALT enzyme levels were stronger for models LPF_2d, LPF_3d, and LPF_4d than for LPF_1d (*Fig. 1*). Next, a sensitivity analysis was performed for the detection of LPF. In this analysis, the highest area under the curve was obtained for LPF_2d (0.738) compared with LPF_3d (0.692) and LPF_4d (0.665; *Fig. S2*). LPF_2d was chosen to define LPF in all subsequent analyses.

### Association of LPF with other main postoperative outcomes

**Fig. 2:**
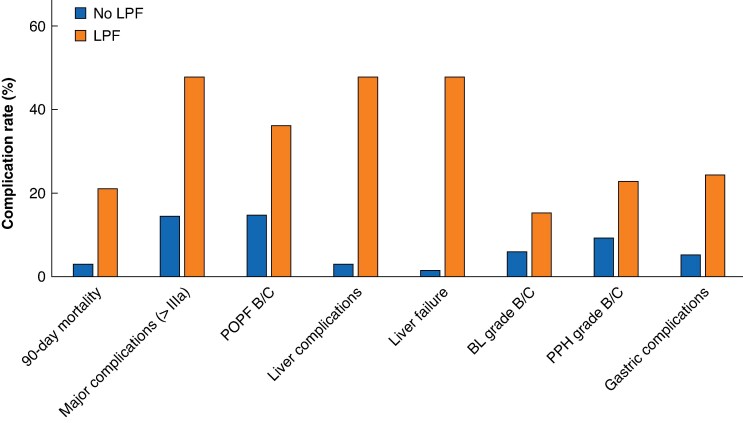
Comparison of clinical outcomes between patients with moderate/severe LPF_2d and those without LPF Major complications were defined as those greater than Clavien–Dindo Grade IIIa. LPF, liver perfusion failure; LPF_2d, liver perfusion failure (alanine aminotransferase/aspartate aminotransferase elevation > 200 U/l) on two consecutive days; POPF, postoperative pancreatic fistula; BL, biliary leakage; PPH, postpancreatectomy haemorrhage. *P < 0.05, **P < 0.001 compared with no liver failure.

One-hundred and three patients were excluded from the analyses for LPF_2 due to lack of complete clinical data and outcomes of 815 patients were used to evaluate the postoperative outcomes. Patients with moderate/severe LPF according to LPF_2d had a significantly higher 90-day mortality rate (11 (21.2%) *versus* 21 (2.9%); *P* < 0.001) and higher rates of major complications (25 (48.1%) *versus* 107 (14.6%); *P* < 0.001), liver-specific complications (21 (40.4%) *versus* 21 (2.9%); *P* < 0.001), liver failure (16 (30.8%) *versus* 12 (1.6%); *P* < 0.001), POPF grade B/C (12 (36.4%) *versus* 89 (14.3%); *P* = 0.001), PPH grade B/C (12 (23.1%) *versus* 68 (9.3%); *P* = 0.001), biliary leakage (8 (15.4%) *versus* 44 (6%); *P* < 0.001), and gastric complications (12 (24.5%) *versus* 38 (5.2%); *P* < 0.001) than patients without LPF (*Table 2* and *Fig. 2*). In most patients, an increase in serum AST/ALT levels was observed before the onset of complications such as POPF or PPH.

**Table 2:** Association of postoperative outcomes with the presence and degree of LPF_2d

	Total (*n* = 815)	LPF_2d					P*
		None (*n* = 734)	Mild (*n* = 29)	Moderate (*n* = 29)	Severe (*n* = 23)	Moderate/severe (*n* = 52)	
**Liver complications‡**	43 (4.3%)	21 (2.9%)	1 (3.4%)	8 (27.6%)	13 (56.5%)	21 (40.4%)	< 0.001
Liver failure	29 (3.6%)	12 (1.6%)	1 (3.4%)	7 (24.1%)	9 (39.1%)	16 (30.8%)	< 0.001
Parenchymal damage	28 (3.4%)	13 (1.8%)	2 (6.9%)	5 (17.2%)	8 (34.8%)	13 (25.0%)	< 0.001
**Postoperative pancreatic fistula §**	174 (25.3%)	156 (25.0%)	4 (13.8%)	6 (33.3%)	8 (53.3%)	14 (42.4%)	0.030
Grade B	55 (8.0%)	53 (8.5%)	0 (0.0%)	1 (5.6%)	1 (6.7%)	2 (6.1%)	0.408
Grade C	47 (6.8%)	36 (5.8%)	1 (3.4%)	3 (16.7%)	7 (46.7%)	10 (30.3%)	< 0.001
Grade B/C	102 (14.8%)	89 (14.3%)	1 (3.4%)	4 (22.3%)	8 (53.4%)	12 (36.4%)	0.001
**Postpancreatectomy haemorrhage**	93 (11.4%)	79 (10.8%)	1 (3.4%)	7 (24.1%)	6 (26.1%)	13 (25.0%)	0.001
Grade B	42 (5.2%)	38 (5.2%)	0 (0.0%)	3 (10.3%)	1 (4.3%)	4 (7.7%)	0.394
Grade C	39 (4.8%)	30 (4.1%)	1 (3.4%)	3 (10.3%)	5 (21.7%)	8 (15.4%)	< 0.001
Grade B/C	81 (9.9%)	68 (9.3%)	1 (3.4%)	6 (20.7%)	6 (26.1%)	12 (23.1%)	0.001
**Biliary leakage**							
Grade B/C	55 (6.7%)	44 (6.0%)	3 (10.3%)	3 (10.3%)	5 (21.7%)	8 (15.4%)	< 0.001
Gastric complications	51 (6.3%)	38 (5.2%)	1 (3.4%)	7 (24.1%)	5 (21.7%)	12 (23.1%)	< 0.001
Major complications¶	139 (17.1%)	107 (14.6%)	7 (24.1%)	12 (41.4%)	13 (56.5%)	25 (48.1%)	< 0.001
90-day mortality	35 (4.3%)	21 (2.9%)	3 (10.3%)	5 (17.2%)	6 (26.1%)	11 (21.2%)	< 0.001
ICU/IMCU stay (days), median (i.q.r.)	2 (1–5)	2 (2–3)	3 (3–4)	5 (4–6)	11 (5–16)	6 (4–10)	< 0.001†
LOS (days), median (i.q.r.)	14 (10–22)	14 (14–15)	15 (13–24)	20 (14–31)	25 (16–50)	22 (16–31)	< 0.001†

Values are *n* (%) unless otherwise specified. *P* values for comparison between patients with and without LPF_2d moderate/ severe. ‡Patients could have both liver parenchymal damage and acute liver failure, but were recorded as a liver-specific complication only once. §Only patients who underwent pancreatoduodenectomy (687) were at risk and were included in this analysis. ¶Major complications followed the definition of Dindo *et al*.^23^. LPF_2d, liver perfusion failure (alanine aminotransferase/aspartate aminotransferase elevation > 200 U/l) on two consecutive days; ICU, intensive care unit; IMCU, intermediate care unit, i.q.r., interquartile range; LOS, length of hospital stay. *χ^2^ test, except †Student’s *t* test.

### Perioperative risk factors for LPF

Potential risk factors for moderate/severe LPF according to LPF_2d were investigated. Univariable testing showed that American Society of Anesthesiologists grade (*P* = 0.001), CAS (≥ 50%) on preoperative CT imaging (*P* < 0.001), resection of the coeliac trunk and/or the hepatic artery (*P* < 0.001), venous resections (*P* < 0.001), intraoperative blood loss (*P* < 0.001), duration of the operation (*P* < 0.001), and partial pancreatoduodenectomy (*P* < 0.001) were significantly associated with LPF (*Table 3*). Liver artery anatomy^21^ was tested as a potential factor for LPF. However, the existence of aberrant or accessory liver arteries was not significantly associated with the occurrence of LPF in this cohort (*Table 3*).

**Table 3:** Perioperative risk factors associated with the incidence of LPF_2d

	Total	LPF_2d			
		Mild (≥ 200 U/L)	Moderate (≥ 500 U/L)	Severe (≥ 1000 U/L)	Moderate and severe (≥ 500 U/L)
Arterial resection (CT or HA)	< 0.001	0.053	< 0.001	< 0.001	< 0.001
Venous resection (SMV or PV)	< 0.001	0.072	< 0.001	0.933	0.002
Intraoperative blood loss	< 0.001	< 0.001	0.005	0.002	< 0.001
Operation time	< 0.001	< 0.001	0.017	< 0.001	< 0.001
Partial PD*	< 0.001	0.006	0.002	0.004	< 0.001
Preoperative ALT levels	< 0.001	< 0.001	< 0.001	0.053	0.011
Coeliac axis stenosis (total)	0.001	0.132	0.017	0.135	0.005
Accessory liver arteries† (V, VI, VII, VIII)	0.239	0.452	0.530	0.577	0.393
Aberrant liver arteries† (II, III, IV, IX)	0.853	0.642	0.746	0.916	0.862
Aberrant/accessory liver arteries†	0.837	0.842	0.606	0.936	0.656

Values show *P* values for each of the factors evaluated. Significance was set at *P* < 0.05. *Only patients who underwent partial PD (*n* = 687) were at risk and included in this analysis. †Anatomy of the liver and its variant blood supply and collateral circulation as per Michels^21^. LPF_2d, liver perfusion failure (ALT/AST elevation > 200 U/l) for two consecutive postoperative days; ALT, alanine aminotransferase; AST, aspartate aminotransferase; CT, coeliac trunk; HA, right/left hepatic artery; SMV, superior mesenteric vein; PV, portal vein; PD, pancreatoduodenectomy.

Multivariable analysis identified arterial resection of the coeliac trunk and/or the hepatic artery (*P* < 0.001), procedure duration (*P* = 0.020), and CAS (*P* < 0.001) as independent predictors for LPF (*Table 4*).

**Table 4:** Multivariable analysis of perioperative risk factors associated with the incidence of liver perfusion failure (ALT/AST elevation > 200 U/l) on two consecutive days, excluding preoperative ALT levels ≥ 500 U/l

	RC	s.e.	Wald	d.f.	*P*	Exp(B)
Partial PD *versus* TP	−0.514	0.363	2.009	1	0.156	0.598
Arterial resection (coeliac trunk and/or hepatic artery)	2.026	0.461	19.313	1	< 0.001	7.585
Venous resection (SMV or PV)	0.129	0.366	0.125	1	0.724	1.138
Intraoperative blood loss	0.000	0.000	1.216	1	0.270	1.000
Operation time (min)	0.004	0.002	5.408	1	0.020	1.004
Coeliac axis stenosis (50–100%)	1.657	0.384	18.624	1	< 0.001	5.244

Variables from univariable analysis were considered for multivariable modelling; selection was based on clinical relevance and collinearity. ALT, alanine aminotransferase; AST, aspartate aminotransferase; RC, regression coefficient; s.e., standard error; d.f., degrees of freedom; PD, pancreatoduodenectomy; TP, total pancreatoduodenectomy; SMV, superior mesenteric vein; PV, portal vein; min, minutes.

## Discussion

LPF is a rarely reported but potentially life-threatening complication after pancreatoduodenectomy. The lack of a clinically relevant and commonly accepted definition may result in underreporting and underrating of LPF. This study established a new prediction model for LPF based on elevated AST and ALT levels for two or more consecutive postoperative days in patients undergoing partial PD or TP that was significantly associated with both liver-specific complications and other main postoperative outcomes defining the course after pancreatic surgery.

This study analysed a large cohort of patients who underwent partial PD or TP and found that mild increases in AST/ALT (200–499 U/l) for two consecutive days were generally not associated with poor surgical outcomes. Moderate (AST/ALT ≥ 500 U/l) and severe (AST/ALT ≥ 1000 U/l) increases in AST/ALT levels were associated with significantly increased liver- and pancreas-specific complications and resulted in higher overall morbidity and mortality. Mild increases in AST/ALT can be influenced by various perioperative factors, such as drug administration and intraoperative manipulation^29,30^. In contrast, the reasons for moderate to severe AST/ALT increases are more complex, and may frequently be associated with irreversible tissue damage, and need thorough investigation, as well as subsequent interventions, as shown in this study. In this context, the postoperative increase in AST/ALT likely reflects early ischaemic liver injury, with surgical complexity, characterized by arterial resection, CAS, and prolonged operative time, a direct risk factor for impaired hepatic perfusion. Moderate and severe LPF as defined by the LPF_2d model were significantly associated with severe clinical outcomes, including liver failure, intensive care unit admission, the need for radiological interventions, and 90-day mortality.

Different outcome parameters were tested to identify risk factors for LPF, including CAS as a potential effector of liver perfusion failure^22,31–33^. CAS was observed in 256 patients (27.9%), a higher incidence than typically reported, likely due to the systematic re-evaluation of CT scans using sagittal reconstructions. CAS was identified as one of three independent risk factors leading to moderate and severe LPF. Of note, 59.3% of patients with CAS were not identified before surgery and were only detected intraoperatively or during this retrospective analysis, indicating that the significance of CAS is still underestimated as a risk factor for perioperative outcomes, including LPF and POPF^12,33^.

Arterial resection in pancreatic surgery is associated with increased morbidity and mortality rates, even in high-volume centres^34^. As expected, resection of the hepatic artery and coeliac trunk were also identified as independent risk factors for LPF. Consequently, patients with true infiltration of the arterial wall who undergo arterial resection require even closer postoperative monitoring to prevent severe complications and, with increasing use of arterial resections for locally advanced pancreatic cancers, the incidence of clinically relevant LPF is expected to rise. At the Department of General, Visceral and Transplantation Surgery, Heidelberg University Hospital, patients undergoing vascular resection receive individualized thromboprophylaxis based on intraoperative findings and vascular reconstruction type^35,36^. Early postoperative Doppler ultrasound is performed within the first 2 hours after surgery with venous or arterial reconstructions. Cross-sectional imaging with contrast-enhanced CT is reserved for patients with clinical signs of hepatic dysfunction or systemic deterioration. These measures aim to ensure the early detection and management of vascular complications potentially contributing to LPF. Conversely, arterial divestment, especially in the setting of neoadjuvant therapy, should be the preferred option to clear the vessels because it is linked to lower complications, as reported previously^34^. In addition to these vascular-related risk factors, the duration of the procedure was identified as an independent risk factor for LPF. A longer operation time is known to be associated with worse perioperative outcomes after pancreatic surgery which can be reduced through higher expertise^37^. Technical advances have shortened the overall operation time; however, increased complexity and oncological radicality have increased in past years, requiring a longer operation time^38,39^. Minimizing operation time through precise procedure planning and performing pancreatic surgery in high-volume centres may therefore improve clinical outcomes and reduce the risk of LPF^40^. Although the primary focus of this study was on anatomical and surgical risk factors for LPF, it should be noted that preoperative chemotherapy may also contribute to hepatic injury. Modern multiagent regimens, such as FOLFIRINOX (folinic acid, fluorouracil, irinotecan, oxaliplatin), include compounds like oxaliplatin and irinotecan, which are known to induce hepatic injury and steatohepatitis^41^.

A previous study by Hackert *et al*.^5^ suggested that preoperative jaundice or cholangitis could affect the incidence of LPF. The present study validated this association and showed that pre-existing cholangitis in patients with pancreatic head tumours usually quickly resolves after partial PD or TP. A sensitivity analysis excluding patients with preoperative elevated ALT levels (≥ 500 U/l) indicated that high preoperative ALT levels increase the rate of mild LPF but are not the main reason for moderate and severe LPF as per the LPF_2d definition. This analysis showed a significant association between LPF and POPF. Although causality remains unclear, early hepatic ischaemia may impair liver function and the immune response, predisposing patients to POPF. Conversely, POPF and its systemic effects may aggravate hepatic injury^42^. This interaction highlights the need for early monitoring in high-risk patients, particularly after complex resections.

This study has several limitations. The retrospective design may have introduced confounding and associations between LPF, major postoperative complications, higher Clavien–Dindo grades, and intensive care unit admission should be interpreted as markers of surgical complexity and postoperative severity rather than a direct cause–effect relationship. The relatively small sample size in subgroups such as patients with aberrant or accessory hepatic arteries may limit generalizability. The proposed AST/ALT-based definition of LPF has not been externally validated. Other chronic liver disease and thrombotic complications were not systematically recorded, limiting assessment of their potential contribution to LPF, particularly after vascular reconstruction.

LPF should be closely monitored and reported in patients undergoing partial PD or TP, because it is a clinically relevant postoperative complication with significantly increased morbidity and mortality. Preoperative radiological imaging can help detect risk factors for LPF, such as CAS. Patients after partial PD with arterial resections are at increased risk of LPF and should be specifically monitored for LPF. It is recommended ALT/AST levels are closely monitored, especially in the early postoperative course, with special attention to levels ≥ 500 U/l. Stable patients with mild LPF can be managed conservatively, whereas patients with more severe LPF may require additional interventions.

## Supplementary Material

zrag056_Supplementary_Data

## Data Availability

Study data are available upon reasonable request to the corresponding author.
