# Supplementary material for: Liver perfusion failure after pancreatoduodenectomy: clinical significance and perioperative risk factors
Source: BJS Open. 2026 Jul 21;10(4):zrag056. doi: 10.1093/bjsopen/zrag056 (PMC13387168; doi:10.1093/bjsopen/zrag056)
Supplement: zrag056_Supplementary_Data [file zrag056_supplementary_data.docx]

**Liver Perfusion Failure After Pancreatoduodenectomy: Clinical Significance and Perioperative Risk Factors**

Mohammed Al-Saeedi^1^, Julian M. Deisenhofer1, Hendrik B. Sauer^1^, Leonie Frank-Moldzio^1^, Alina S. Ritter^1^, Tom Bruckner^2^, Gabriel A. Salg^1^, Ali Ramouz^1^, Philipp Mayer^3^, Thomas Hank^1^, Arianeb Mehrabi^1^, Martin Loos^1^, Thilo Hackert^1^, Markus W. Büchler^1,4^ and Oliver Strobel^1,5^

^1^Department of General, Visceral and Transplantation Surgery, Heidelberg University Hospital, Heidelberg, Germany

^2^Institute of Medical Biometry and Informatics, Heidelberg University Hospital, Heidelberg, Germany

^3^Department of Diagnostic and Interventional Radiology, Heidelberg University Hospital, Heidelberg, Germany

^4^Botton‒Champalimaud Pancreatic Cancer Centre, Lisbon, Portugal

^5^Division of Visceral Surgery, Department of General Surgery, Medical University of Vienna, Vienna, Austria

**Corresponding author and request for reprints:**

Oliver Strobel, MD

Department of General, Visceral and Transplantation Surgery

Heidelberg University Hospital

Im Neuenheimer Feld 420

69120 Heidelberg, Germany

Phone: +49 6221 566110

Fax: +49 6221 565969

E-Mail: ostrobel@gmx.de

**Supplementary Materials - Index**

| **Supplementary Figures and Tables** |  | |
| --- | --- | --- |
| Suppl. Figure 1 Study flow chart  Suppl. Figure 2 Prognostic accurancy of different LPF models  Suppl. Table 1 Overview for LPF using different identification models | | *pag. 3*  *pag. 4*  *pag. 5* |
| Suppl. Table 2 a-c | *pag. 6-8* | |
|  |  | |

**Supplementary Figures and Tables**

**
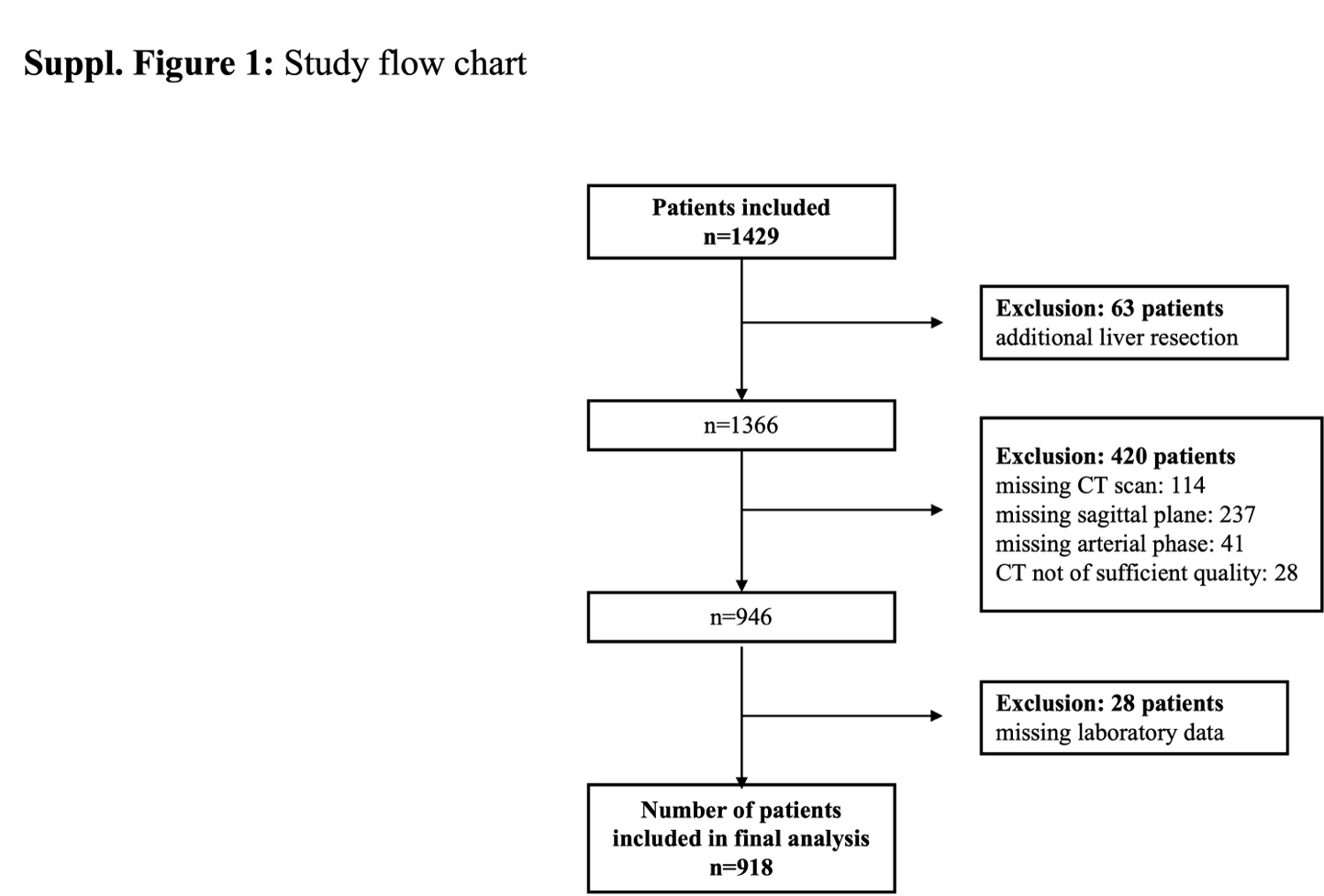
**

**
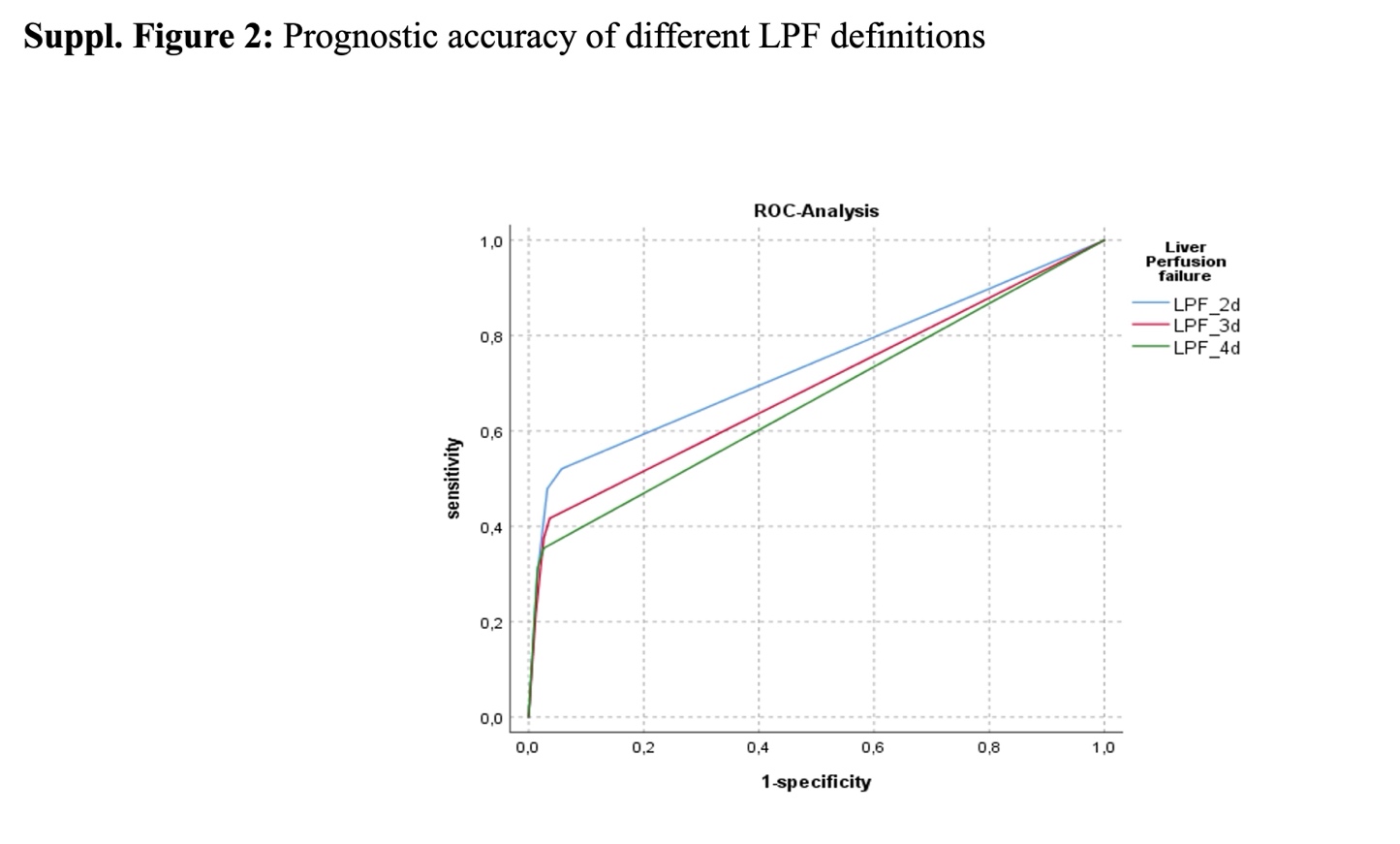
**

| **Suppl. Table 1:** Overview for LPF using different identification models | | | | |
| --- | --- | --- | --- | --- |
| Identification method | laboratory data | n=918(%) | |  |
| LPF_1d* | combined (AST/ALT) | 176 | (19.2%) | |
|  | mild (POD 1 and 3 ≥250-499U/L) | 60 | (6.5%) | |
|  | moderate (POD 1 and 3 500-999U/L) | 95 | (10.4%) | |
|  | severe (POD 1 and 3 ≥1000U/L) | 21 | (2.3%) | |
|  | moderate & severe (POD 1 and 3 ≥500U/L) | 116 | (12.6%) | |
| LPF_2d* | combined (AST/ALT) | 110 | (12.0%) | |
|  | mild (≥200 - 499U/L) | 48 | (5.2%) | |
|  | moderate (≥500- 999U/L) | 35 | (3.8%) | |
|  | severe (≥1000 U/L) | 27 | (2.9%) | |
|  | moderate & severe (≥500U/L) | 62 | (6.8%) | |
|  |  |  |  | |
| LPF_3d * | combined (AST/ALT) | 67 | (7.3%) | |
|  | mild (≥200 - 499U/L) | 15 | (1.6%) | |
|  | moderate (≥500- 999U/L) | 30 | (3.3%) | |
|  | severe (≥1000 U/L) | 22 | (2.4%) | |
|  | moderate & severe (≥500U/L) | 52 | (5.7%) | |
|  |  |  |  | |
| LPF_4d * | combined (AST/ALT) | 44 | (4.8%) | |
|  | mild (≥200 - 499U/L) | 5 | (0.5%) | |
|  | moderate (≥500- 999U/L) | 20 | (2.2%) | |
|  | severe (≥1000 U/L) | 19 | (2.1%) | |
|  | moderate & severe (≥500U/L) | 39 | (4.3%) | |
| *Liver Perfusion Failure (LPF) defined by AST/ALT elevation for 1d or a period of 2,3,4 consecutive days after pancreatoduodenectomy. | | | |  |

| **Suppl. Table 2a:** Liver Perfusion Failure Data Overview (combined ALT & AST) | | | | | | | | | | |
| --- | --- | --- | --- | --- | --- | --- | --- | --- | --- | --- |
|  |  | n(918) | %(n) | %(sub) | LSC  (n=54) | | TD  (n=32) | | LF  (n=33) | |
| LPF_1d | combined (ALT/AST) | 176 | 19.2% | 100.00% | 30 | 55.6% | 16 | 50.0% | 18 | 54.6% |
|  | mild (POD 1 and 3 ≥250-499U/L) | 60 | 6.5% | 34.1% | 2 | 3.7% | 0 | 0.0% | 2 | 6.1% |
|  | moderate (POD 1 and 3 500-999U/L) | 95 | 10.4% | 54.0% | 16 | 29.6% | 9 | 28.1% | 9 | 27.3% |
|  | severe (POD 1 and 3 ≥1000U/L) | 21 | 2.3% | 11.9% | 12 | 22.2% | 7 | 21.9% | 7 | 21.2% |
|  | mod. + sev. (POD 1 and 3 ≥500U/L) | 116 | 12.6% | 65.9% | 28 | 51.9% | 16 | 50.0% | 16 | 48.5% |
|  |  |  |  |  |  |  |  |  |  |  |
| LPF_2d | combined (ALT/AST) | 110 | 12.0% | 100.0% | 29 | 53.7% | 16 | 50.0% | 18 | 54.6% |
|  | mild (≥200-499 U/L) | 48 | 5.2% | 43.6% | 3 | 5.6% | 2 | 6.3% | 2 | 6.1% |
|  | Moderate (≥500-999U/L) | 35 | 3.8% | 31.8% | 11 | 20.4% | 5 | 15.6% | 7 | 21.2% |
|  | severe (≥1000 U/L) | 27 | 2.9% | 24.6% | 15 | 27.8% | 9 | 28.1% | 9 | 27.3% |
|  | mod. + sev. (≥500U/L) | 62 | 6.8% | 56.4% | 26 | 48.2% | 14 | 43.8% | 16 | 48.5% |
|  |  |  |  |  |  |  |  |  |  |  |
| LPF_3d | combined (ALT/AST) | 67 | 7.3% | 100.0% | 25 | 46.3% | 12 | 37.5% | 17 | 51.5% |
|  | mild (≥200 499U/L) | 15 | 1.6% | 22.4% | 2 | 3.7% | 1 | 3.1% | 2 | 6.1% |
|  | moderate(≥500-999U/L) | 30 | 3.3% | 44.8% | 12 | 22.2% | 5 | 15.6% | 8 | 24.2% |
|  | severe (≥1000 U/L) | 22 | 2.4% | 32.8% | 11 | 20.4% | 6 | 18.8% | 7 | 21.2% |
|  | mod. + sev. (≥500U/L) | 52 | 5.7% | 77.6% | 23 | 42.6% | 11 | 34.4% | 15 | 45.5% |
|  |  |  |  |  |  |  |  |  |  |  |
| LPF_4d | combined (ALT/AST) | 44 | 4.8% | 100.0% | 20 | 37.0% | 9 | 28.1% | 14 | 42.4% |
|  | mild (≥200-499U/L) | 5 | 0.5% | 11.4% | 1 | 1.9% | 0 | 0.0% | 9 | 27.3% |
|  | moderate(≥500-999U/L) | 20 | 2.2% | 45.45% | 9 | 16.7% | 3 | 9.4% | 8 | 24.2% |
|  | severe (≥1000 U/L) | 19 | 2.1% | 43.2% | 10 | 18.5% | 5 | 15.7% | 6 | 18.2% |
|  | mod. + sev. (≥500U/L) | 39 | 4.3% | 88.6% | 19 | 35.2% | 8 | 25.0% | 14 | 42.4% |

LSC, liver specific complications; TD, tissue damage; LF, liver failure

| **Suppl. Table 2b:** Liver Perfusion Failure Data Overview (ALT) | | | | | | | | | | | | |
| --- | --- | --- | --- | --- | --- | --- | --- | --- | --- | --- | --- | --- |
|  |  | n(918) | %(n) | %(sub) | LSC  (n=54) | | TD  (n=32) | | LF  (n=33) | | | |
| LPF_1d | ALT | 162 | 17.7% | 100.0% | 30 | 55.6% | 16 | 50.0% | | 18 | 54.6% |  |
|  | mild (POD 1 and 3 ≥250-499U/L) | 114 | 12.4% | 70.4% | 11 | 20.4% | 5 | 15.6% | | 7 | 21.2% |  |
|  | moderate (POD 1and 3 500-999U/L) | 27 | 2.9% | 16.7% | 7 | 13.0% | 4 | 12.5% | | 4 | 12.1% |  |
|  | severe (POD 1 and 3 ≥1000U/L) | 21 | 2.3% | 13.0% | 12 | 22.2% | 7 | 21.9% | | 7 | 21.2% |  |
|  | mod. + sev. (POD 1 and 3 ≥500U/L) | 48 | 5.2% | 29.6% | 19 | 35.2% | 11 | 34.4% | | 11 | 33.3% |  |
|  |  |  |  |  |  |  |  |  | |  |  |  |
| LPF_2d | ALT | 106 | 11.6% | 100.0% | 29 | 53.7% | 16 | 50.0% | | 18 | 54.6% |  |
|  | mild (≥200-499 U/L) | 60 | 6.5% | 56.6% | 9 | 16.7% | 4 | 12.5% | | 7 | 21.2% |  |
|  | moderate (≥500-999 U/L) | 26 | 2.83% | 24.5% | 9 | 16.7% | 5 | 15.6% | | 5 | 15.2% |  |
|  | severe (≥1000 U/L) | 20 | 2.18% | 18.9% | 11 | 20.4% | 7 | 21.9% | | 6 | 18.2% |  |
|  | mod. + sev.(≥500U/L) | 46 | 5.01% | 43.4% | 20 | 37.0% | 12 | 37.5% | | 11 | 33.3% |  |
|  |  |  |  |  |  |  |  |  | |  |  |  |
| LPF_3d | ALT | 63 | 6.9% | 100.0% | 23 | 42.6% | 12 | 37.5% | | 15 | 45.6% |  |
|  | mild (≥200-499 U/L) | 22 | 2.4% | 34.9% | 6 | 11.1% | 3 | 9.4% | | 5 | 15.2% |  |
|  | moderate (≥500-999 U/L) | 24 | 2.6% | 38.1% | 8 | 14.8% | 4 | 12.5% | | 5 | 15.2% |  |
|  | severe (≥1000 U/L) | 18 | 2.0% | 28.6% | 9 | 16.7% | 5 | 15.6% | | 5 | 15.2% |  |
|  | mod. + sev.(≥500U/L) | 42 | 4.6% | 66.7% | 17 | 31.5% | 9 | 28.1% | | 10 | 30.3% |  |
|  |  |  |  |  |  |  |  |  | |  |  |  |
| LPF_4d | ALT | 41 | 4.5% | 100.0% | 18 | 33.33% | 9 | 28.1% | | 12 | 36.4% |  |
|  | mild (≥200-499 U/L) | 7 | 0.8% | 17.1% | 4 | 7.4% | 2 | 6.3% | | 3 | 9.1% |  |
|  | moderate (≥500-999 U/L) | 17 | 1.9% | 41.5% | 5 | 9.3% | 2 | 6.3% | | 4 | 12.1% |  |
|  | severe (≥1000 U/L) | 17 | 1.9% | 41.5% | 9 | 16.7% | 5 | 15.6% | | 5 | 15.2% |  |
|  | mod. + sev. (≥500U/L) | 34 | 3.7% | 82.9% | 14 | 25.9% | 7 | 21.9% | | 9 | 27.3% |  |

LSC, liver specific complications; TD, tissue damage; LF, liver failure

| **Suppl. Table 2c:** Liver Perfusion Failure Data Overview (AST) | | | | | | | | | | |
| --- | --- | --- | --- | --- | --- | --- | --- | --- | --- | --- |
|  |  | n(918) | %(n) | %(sub) | LSC  (n=54) | | TD  (n=32) | | LF  (n=33) | |
| LPF_1d | AST | 173 | 18.9% | 100.0% | 18 | 33.3% | 16 | 50.0% | 18 | 54.6% |
|  | mild (POD 1 and 3 ≥250-499U/L) | 61 | 6.6% | 35.3% | 2 | 3.7% | 0 | 0.0% | 2 | 6.1% |
|  | moderate (POD 1and 3 500-999U/L) | 91 | 9.9% | 52.6% | 4 | 7.4% | 9 | 2.1% | 9 | 27.3% |
|  | severe (POD 1 and 3 ≥1000U/L) | 21 | 2.3% | 12.1% | 12 | 22.2% | 7 | 21.9% | 7 | 21.2% |
|  | mod. + sev. (POD 1 and 3 ≥500U/L) | 112 | 12.2% | 64.7% | 16 | 29.6% | 16 | 50.0% | 16 | 48.5% |
|  |  |  |  |  |  |  |  |  |  |  |
| LPF_2d | AST | 71 | 7.7% | 100.0% | 26 | 48.2% | 14 | 43.8% | 16 | 48.5% |
|  | mild (≥200-499 U/L) | 16 | 1.7% | 22.5% | 1 | 1.9% | 1 | 3.1% | 0 | 0.0% |
|  | moderate (≥500-999 U/L) | 28 | 3.1% | 39.4% | 10 | 18.5% | 4 | 12.5% | 7 | 21.2% |
|  | severe (≥1000 U/L) | 27 | 2.9% | 38.0% | 15 | 27.8% | 9 | 28.1% | 9 | 27.3% |
|  | mod. + sev.(≥500U/L) | 55 | 6.0% | 77.5% | 25 | 46.3% | 13 | 40.6% | 16 | 48.5% |
|  |  |  |  |  |  |  |  |  |  |  |
| LPF_3d | AST | 41 | 4.5% | 100.0% | 20 | 37.0% | 10 | 31.3% | 13 | 39.4% |
|  | mild (≥200-499 U/L) | 4 | 0.4% | 9.8% | 0 | 0.0% | 0 | 0.0% | 0 | 0.0% |
|  | moderate (≥500-999 U/L) | 16 | 1.7% | 39.0% | 9 | 16.7% | 4 | 12.5% | 6 | 18.2% |
|  | severe (≥1000 U/L) | 21 | 2.3% | 51.2% | 11 | 20.4% | 6 | 18.8% | 7 | 21.2% |
|  | mod. + sev. (≥500U/L) | 37 | 4.3% | 90.2% | 20 | 37.0% | 10 | 31.3% | 13 | 39.4% |
|  |  |  |  |  |  |  |  |  |  |  |
| LPF_4d | AST | 20 | 2.2% | 100.0% | 12 | 22.2% | 6 | 18.8% | 8 | 24.2% |
|  | mild (≥200-499 U/L) | 2 | 0.2% | 10.0% | 0 | 0.0% | 0 | 0.0% | 0 | 0.0% |
|  | moderate (≥500-999 U/L) | 6 | 0.7% | 30.0% | 5 | 9.3% | 2 | 6.3% | 4 | 12.1% |
|  | severe (≥1000 U/L) | 12 | 1.3% | 60.0% | 7 | 13.0% | 4 | 12.5% | 4 | 12.1% |
|  | mod. + sev. (≥500U/L) | 18 | 2.0% | 90.0% | 12 | 22.2% | 6 | 18.8% | 8 | 24.2% |

LSC, liver specific complications; TD, tissue damage; LF, liver failure
